# Pharmacoepidemiologic Research Based on Common Data Models: Systematic Review and Bibliometric Analysis

**DOI:** 10.2196/72225

**Published:** 2025-07-28

**Authors:** Yongqi Zheng, Meng Zhang, Conghui Wang, Ling Gao, Junqing Xie, Peng Shen, Yexiang Sun, Mengling Feng, Seng Chan You, Feng Sun

**Affiliations:** 1 Department of Epidemiology and Biostatistics, School of Public Health, Peking University, 38 Xueyuan Road, Haidian District, Beijing, 100191, China, 86 13811155891; 2 NDORMS, The Botnar Research Centre, University of Oxford, Oxford, United Kingdom; 3 Yinzhou District Center for Disease Control and Prevention, Ningbo, China; 4 Saw Swee Hock School of Public Health and the Institute of Data Science, National University of Singapore, Singapore, Singapore; 5 Department of Biomedical Systems Informatics, Yonsei University College of Medicine, Seoul, Republic of Korea; 6 Institute of Traditional Chinese Medicine, Xinjiang Medical University, Urumqi, 830017, China; 7 Department of Ophthalmology, Peking University Third Hospital, Beijing, 100191, China; 8 School of Medicine, Shihezi University, Shihezi, 832000, China

**Keywords:** common data model, pharmacoepidemiology, bibliometric analysis, systematic review

## Abstract

**Background:**

The adoption of common data models (CDMs) has transformed pharmacoepidemiologic research by enabling standardized data formatting and shared analytical tools across institutions. These models facilitate large-scale, multicenter studies and support timely real-world evidence generation. However, no comprehensive global evaluation of CDM applications in pharmacoepidemiology has been conducted.

**Objective:**

This study aimed to conduct a systematic review and bibliometric analysis to map the landscape of CDM usage in pharmacoepidemiology, including publication trends, institutional authors and collaborations, and citation impacts.

**Methods:**

In total, 5 English databases (PubMed, Web of Science, Embase, Scopus, and Virtual Health Library) and 4 Chinese databases (CNKI, Wan-Fang Data, VIP, and SinoMed) were searched for studies applying CDMs in pharmacoepidemiology from database inception to January 2024. Two reviewers independently screened studies and extracted information about basic publication details, methodological details, and exposure and outcome information. The studies were categorized into 2 groups according to their Total Citations per Year (TCpY), and a comparative analysis was conducted to examine the differences in characteristics between the 2 groups.

**Results:**

A total of 308 studies published between 1997 and 2024 were included, involving 1580 authors across 32 countries and 140 journals. The United States led in both publication volume and citation counts, followed by South Korea. Among the 10 most cited studies, 7 used the Vaccine Safety Datalink, 2 used Sentinel, and one used Observational Medical Outcomes Partnership. Studies were stratified by TCpY to reduce citation bias from publication timing. Comparative analysis showed that high-TCpY studies were significantly more associated with multicenter collaboration (*P*=.008), United States-based institutions (*P*=.04), and vaccine-related research (*P*=.009). These studies commonly featured larger sample sizes, cross-regional data, and enhanced generalizability. International collaborations primarily occurred among North America, Europe, and East Asia, with limited involvement from limited-income countries.

**Conclusions:**

This study presents the first bibliometric overview of CDM-based pharmacoepidemiologic research. The consistent output from United States institutions and increasing engagement from South Korea underscore their central roles in this field. High-TCpY studies tend to be multicenter, collaborative, and vaccine-focused, reflecting structural factors linked to research visibility and influence. Stratified citation analysis supports the value of real-world data integration and international cooperation in producing impactful studies. The dominance of limited-income countries in collaboration networks highlights a need for broader inclusion of underrepresented regions. These findings can help researchers identify key contributors, guide partner selection, and target appropriate journals. As CDM-based methods continue to expand, fostering diverse and collaborative research efforts will be crucial for advancing pharmacoepidemiologic knowledge globally.

## Introduction

Pharmacoepidemiology applies epidemiological principles and methods to study the effects of drugs, vaccines, and devices, focusing on adverse event monitoring, health economic evaluation, and quality of life assessment [1]. Ongoing pharmacoepidemiologic research aims to ensure drug safety, standardize postmarket surveillance methods, and provide scientific evidence for intervention decisions [1]. To achieve these objectives, especially in postmarketing contexts, robust real-world evidence is essential. However, many current studies are limited by small sample sizes and insufficient population representativeness, making it difficult to detect rare or long-term adverse events [2]. To address these limitations, conducting multicenter pharmacoepidemiologic evaluations has proven to be more reflective of actual clinical settings, enhancing statistical power, improving detection efficiency, and enabling early identification of vaccine safety concerns [3,4]. However, multicenter studies also face challenges related to data heterogeneity, personally identifiable information leakage, lack of standardized protocols for data integration, and terminological harmonization difficulties [5,6].

Common data model (CDM) is a standardized data model designed to facilitate the exchange, integration, sharing, or storage of data from multiple sources [7]. Its development has provided a practical and scalable solution for enabling multicenter studies by harmonizing heterogeneous data sources across institutions and regions. Currently, various CDMs are widely used, including the Observational Medical Outcomes Partnership (OMOP) developed by the Observational Health Data Sciences and Informatics [8], PCORnet [9], and Fast Healthcare Interoperability Resources [10]. Other CDMs, such as the Vaccine Safety Datalink (VSD) [11] and Sentinel [12], are specifically designed for active vaccine safety monitoring. Each CDM emphasizes distinct aspects, with differing methodologies and application areas tailored to specific research needs [13].

Currently, CDMs are widely applied to generate new evidence in clinical practice and drug selection. Some studies leverage real-world data to produce scalable evidence, aiding in the understanding of population diversity as well as the similarities and differences in clinical characteristics and treatment pathways across regions [14,15]. In addition, certain studies based on the OMOP have introduced a large-scale, comprehensive approach to evaluate the effectiveness and safety of various drugs across the world, offering strong evidence to support clinical drug selection [16,17]. Despite the growing application of CDMs in pharmacoepidemiology, no study has systematically reviewed the global landscape of CDM use in this field across all major models and contexts. Previous reviews have often focused on a single CDM or specific application scenarios [6].

In recent years, bibliometrics has advanced rapidly and is widely used to explore the characteristics of academic publications in specific research fields, including influential countries, journals, institutions, and authors, as well as trends in frequently cited references and keywords [18,19]. Since Garfield’s seminal work in 1955 on identifying the most cited scientific papers in the Institute for Scientific Information Web of Knowledge (now known as Web of Science) database [20,21], numerous scholars have conducted bibliometric analysis comparing highly cited and less cited papers [22-24]. Building on previous studies that highlighted key features of highly cited papers, this study uses bibliometric analysis to systematically characterize publication patterns, collaboration networks, and thematic trends in CDM-based pharmacoepidemiologic research.

This study aimed to fill that gap by systematically reviewing and visually analyzing global literature on CDM-based pharmacoepidemiology. By grouping studies by citation impact, we sought to identify the characteristics of highly cited studies and offer references for future research design, collaboration strategy, and infrastructure development. Our findings aim to inform researchers and policy makers on emerging trends, methodological priorities, and opportunities to enhance the global reach and inclusiveness of CDM-enabled pharmacoepidemiologic studies. The statement of significance is provided inTable 1.

**Table 1.** Statement of significance.

Content	Statement
Problem or issue	There is a critical challenge in integrating heterogeneous, multicenter, real-world data for pharmacoepidemiologic research, which hampers the detection of rare and long-term adverse events and limits the standardization of postmarket surveillance.
What is already known	Previous studies have demonstrated that CDMs^a^ facilitate data standardization and enhance multicenter collaborations in pharmacoepidemiology. However, the global adoption, comparative impact, and evolution of these models remain insufficiently explored.
What this paper adds	This paper provides a comprehensive bibliometric analysis of the application of CDMs in pharmacoepidemiology, revealing publication trends, key contributors, and emerging research themes across different countries. It also identifies significant differences between high and low citation groups, underscoring the pivotal role of multi-center research and CDM-based methodologies.
Who would benefit from the new knowledge in this paper?	Researchers, policymakers, and healthcare practitioners involved in pharmacoepidemiologic studies and health informatics will benefit, as the findings offer insights into effective collaboration, selection of appropriate research models, and strategic dissemination for enhancing drug and vaccine safety monitoring.

a CDM: common data models.

## Methods

### Overview

This study was conducted according to the PRISMA (Preferred Reporting Items for Systematic Reviews and Meta Analyses) guidelines (the PRISMA checklist is provided in Checklist 1) to identify studies that applied CDM in pharmacoepidemiologic research [25].

### Search Strategy

Five English databases: PubMed, Web of Science, Embase, Scopus, and Virtual Health Library, and 4 Chinese databases: China National Knowledge Infrastructure, Wan-Fang Data, VIP Database, and SinoMed were searched from the inception to January 22, 2024. In preliminary searches, the names of currently used CDMs worldwide were identified and used as search terms in both their full and abbreviated forms, in both English and Chinese, including terms like “common data model,” “Observational Health Data Sciences and Informatics,” “Observational Medical Outcomes Partnership,” and “Clinical Data Interchange Standards Consortium,” among others. Logical operators such as “OR” truncation symbols, and subject term matching were applied based on the search rules and syntax of each platform to form search strategies. All search terms were listed in Multimedia Appendix 1.

### Eligibility Criteria

The inclusion criteria were as follows: (1) studies using CDMs (any CDM identified in the preliminary search, with inclusion based on the use of any single model) to address issues in the drug, vaccine, or medical device fields; (2) the study scope included safety, efficacy, usage, and economic evaluations for drugs, vaccines, and medical devices; and (3) drugs, vaccines, or devices must be the primary exposure, research focus, or outcomes of the study.

The exclusion criteria were as follows: (1) studies that did not apply CDMs, or where the CDM was incomplete; (2) studies that did not include drugs, vaccines, or devices as primary exposure or influence factors, or as main research content or outcomes; (3) editorial materials, including letters, editorials, comments, responses, editorial opinions, advertisements, and unpublished studies; (4) duplicate publications; and (5) studies in languages other than Chinese or English.

### Data Extraction

Two reviewers independently screened studies and extracted information about basic publication details, methodological details, and exposure and outcome information. Basic information from the literature was extracted using Excel, including study title, authors, publication year, country, sample size, number of centers, type of CDMs, use of subgroup analysis, whether sensitivity or subgroup analysis was conducted, study exposure, study outcomes, and whether adherence to reporting guidelines. English studies were exported in BibTeX format and merged into a single XLSX file. Some English studies that could not be imported directly into the Bibliometrix package and all of the Chinese studies were exported in XLSX format. These files were modified into compatible tables based on the Bibliometrix data frame structure for analysis.

### Statistical Analysis

Statistical analysis was conducted using R (version 4.4.0; R Core Team). The bibliometrix package was used to generate standard bibliometric indicators, including annual publication trends, total and average citation counts, and H-index values, as well as to construct coauthorship and keyword co-occurrence networks. To ensure consistency and data quality, all bibliographic records were cleaned and standardized before analysis. This process included deduplication, harmonization of author names and affiliations, normalization of journal titles, and manual review of keywords [26]. Total citation, Total Citations per Year (TCpY), and the H-index were used as standard metrics for academic influence of studies and authors. The total citation of all studies published in a specific journal was used to represent the journal’s overall citation count [27]. TCpY was defined as the average number of citations a study has received per year since its publication, calculated as follows:


$$TCpY\;=\;\frac{Total\;\;Citations}{Years\;\;since\;\;Publication}$$

The H-Index is defined as *h* if an author has *h* publications, each of which has been cited at least *h* times [28]. Citation analysis was conducted on 285 studies which could get citations from the Web of Science Core Collection (WoSCC). Analysis of these 285 studies was conducted using R version 4.4.0. The *t* test was used to compare means under the assumption of normal distribution, while the Wilcoxon test served data that did not meet normality assumptions. Chi-square or Fisher exact tests were used for categorical variables. A 2-tailed *P* value of <.05 was considered to indicate statistical significance. To visualize interdisciplinary citation relationships, we conducted a dual-map overlay analysis using CiteSpace (version 6.4.1; Chaomei Chen, Drexel University). This method enables the mapping of citation trajectories between disciplines, highlighting the knowledge flow from citing to cited journals. The map was constructed using WoSCC data, with parameters set to the default configuration for dual-map overlays.

## Results

### Study Selection

**Figure 1.**
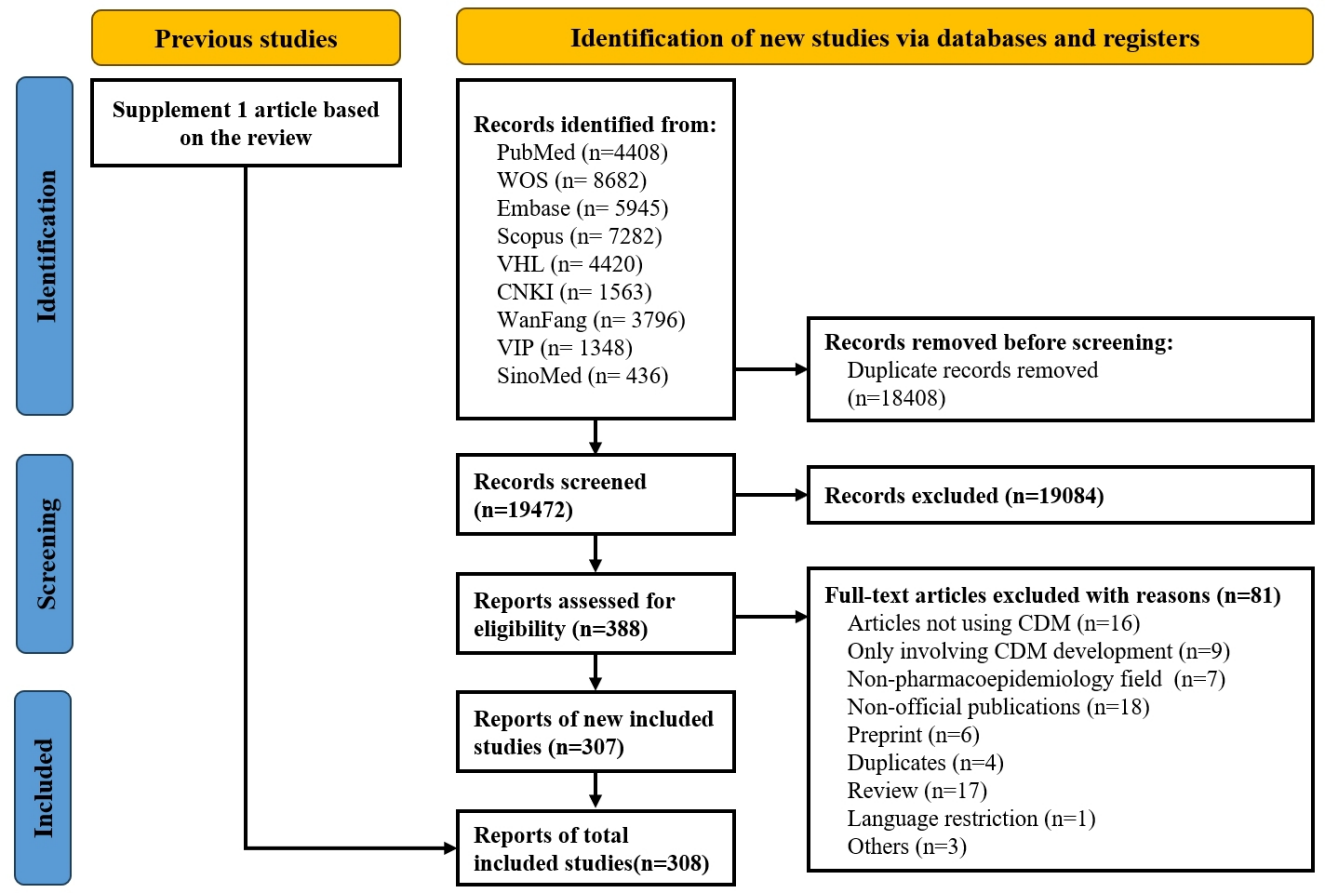
PRISMA (Preferred Reporting Items for Systematic Reviews and Meta-Analyses) flow diagram of the included studies.

A total of 37,880 studies were identified, of which 308 met the inclusion criteria, comprising 307 in English and 1 in Chinese (Figure 1). The publications originated from 32 countries, involving 1580 authors and published across 140 journals. In total, 590 keywords were identified, covering 12 types of CDMs: OMOP (76 studies), VSD (163 studies), PCORnet (14 studies), PEDSnet (4 studies), Sentinel (19 studies), Mini-Sentinel (14 studies), ConcePTION (3 studies), K-CDM (3 studies), Asian Pharmacoepidemiology Network (1 study), Cancer Research Network’s Virtual Data Warehouse (1 study), Intensive Care Unit Medications (1 study), Health Maintenance Organization Research Network (1 study), along with 1 study that modified an existing CDM and 11 studies that created new CDMs. The research topics were categorized as follows: vaccines (173/308, 56.17%), drugs (132/308, 42.85%), and medical devices (3/308, 0.97%).

### Temporal Distribution of Publications

**Figure 2.**
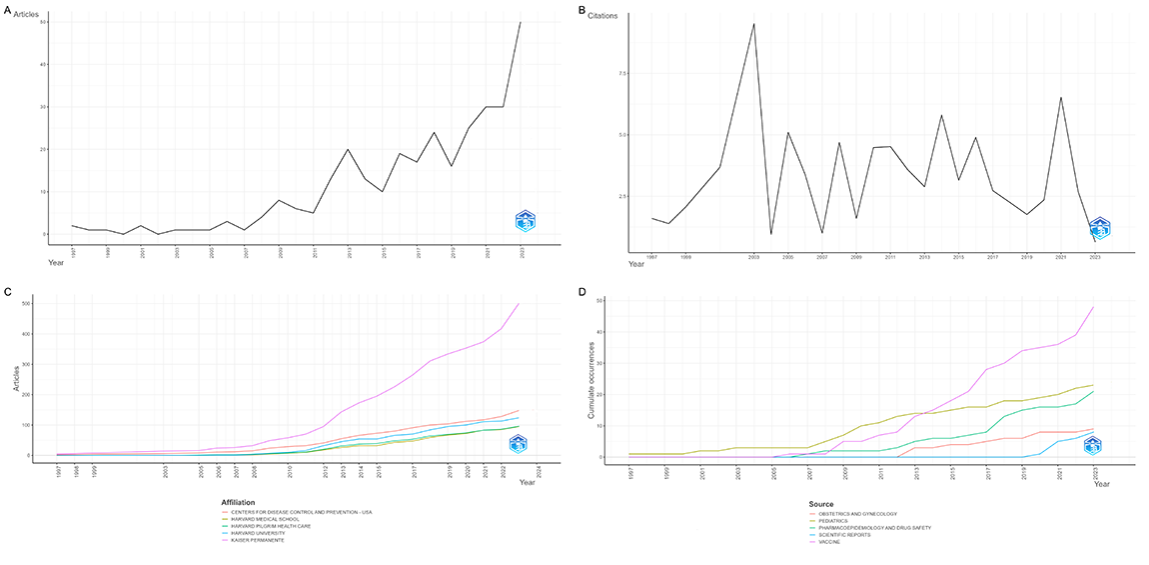
General trends of included studies in CDM-based pharmacoepidemiologic research through 2024. (**A)** Temporal distribution of publications in pharmacoepidemiologic research based on CDM through 2024. (**B)** Average annual citation frequency in CDM-based pharmacoepidemiologic research through 2024. (**C)** Temporal distribution of publications for the top 5 most productive institutions in CDM-based pharmacoepidemiologic research through 2024. (**D)** Annual publication trends across top 5 journals in CDM-based pharmacoepidemiologic research through 2024. CDM: Common Data Model.

The volume of publications in this field has shown a continuous upward trend. The earliest identified study dates back to 1997. From 1997 to 2007, the volume of studies remained low with minimal growth, maintaining fewer than 5 studies per year. Between 2008 and 2016, the number of studies began to increase, albeit with some fluctuation. Following 2019, study volume exhibited steady growth, reaching a peak of 49 studies in 2023 (Figure 2A).

### Citation Information of the Studies

Among the 308 studies, the total citation count was 8666, with an average citation frequency of 28 per study and a median of 13.00 (IQR 3.00‐30.00) citations. The citation distribution by publication year was shown in Figure 2B, with peaks in 2003 and 2021.

The top 10 most cited studies originated entirely from the United States, comprising 7 studies on VSD, 2 on Mini-Sentinel, and one using OMOP. Regarding the focus of exposure, 8 studies investigated vaccines, while 2 examined drugs. Safety outcomes were predominant, accounting for 90% (9/10) of the research endpoints. Multimedia Appendix 2 provides detailed information on the top 10 most cited studies.

The citation analysis was conducted on 285 studies according to citations from the WoSCC. We compared key characteristics between the 285 WoSCC-indexed studies and the 23 non-WoSCC studies. As shown in Multimedia Appendix 3, there were no statistically significant differences between the 2 groups. The included studies were divided into 2 groups based on total citation counts, and their basic characteristics were compared (Table 2). The median citation count for the low citation group was 4.00 (IQR 1.00‐8.00), while for the high-citation group it was 35.00 (IQR 20.00‐64.00). Compared to the low-citation group, studies in the high-citation group generally had larger sample sizes (*P*=.049), involved more research centers (*P*<.001), and predominantly originated from the United States (*P*<.001). Regarding exposure, studies in the high-citation group were more likely to focus on vaccines than those in the low-citation group (72.34%, 102/141 vs 40.97%, 59/144; *P*<.001). A comparison between studies with vaccine as the exposure and those with drug indicated that high-citation studies were more frequently focused on vaccines (*P*<.001). Studies with high citation counts also tended to use the VSD. Differences were observed in research direction, with a higher proportion of safety studies in the high-citation group compared to the low-citation group (75.18%, 106/141 vs 64.58%, 93/144).

**Table 2.** Comparison of characteristics between high and low total citation study groups in CDM^a^-based pharmacoepidemiologic research through 2024.

Variable	Low total citations (N=144)	High total citations (N=141)	*P* value
Total citations, median (IQR)	4 (1-8)	35 (20-64)	<.001
Sample size, median (IQR)	153,438 (10,674-1,600,000)	381,807 (92,357-1,505,381)	.049
Number of centers, median (IQR)	6.00 (3.00-8.00)	7.00 (6.00-8.00)	<.001
Data from the United States, n (%)	88 (61.11)	123 (87.23)	<.001
Research exposure, n (%)			<.001
Drug	83 (57.64)	38 (26.95)	
Vaccine	59 (40.97)	102 (72.34)	
Medical device	2 (1.39)	1 (0.71)	
Types of CDMs n (%)			<.001
OMOP^b^	49 (34.03)	17 (12.06)	
VSD^c^	54 (37.50)	99 (70.21)	
Sentinel or mini-Sentinel	15 (10.42)	14 (9.93)	
Others	26 (18.06)	11 (7.8)	
Research directions, n (%)			.02
Safety	93 (64.58)	106 (75.18)	
Effectiveness	12 (8.33)	2 (1.42)	
Usage	28 (19.44)	19 (13.48)	
Others	11 (7.64)	14 (9.93%)	
Including sensitive analysis, n (%)	46 (32.86)	40 (28.57)	.52
Including subgroup analysis, n (%)	75 (52.45)	54 (38.30)	.02
Including reporting guideline, n (%)	12 (8.33)	4 (2.84)	.07

a CDM: Common Data Model.

b OMOP: Observational Medical Outcomes Partnership.

c VSD: Vaccine Safety Datalink.

We further divided the included studies into 2 groups based on TCpY to compare their characteristics (Table 3). The median annual citation for the low TCpY group was 1.00 (IQR 0.50‐1.50), while for the high TCpY group it was 4.00 (IQR 2.75‐6.28). Compared to the low TCpY group, studies in the high TCpY group generally involved a greater number of research centers (*P*=.008) and were predominantly conducted in the United States (*P*=.045). Regarding exposure, studies in the high TCpY group were more likely to focus on vaccines than those in the low TCpY group (63.70%, 93/146 vs 48.92%, 68/139; *P*=.003). A comparison between studies with vaccine as the exposure and those with drug indicated that high TCpY studies were more frequently focused on vaccines (*P*=.009). The proportion of the high TCpY group using reporting guidelines was higher, although the difference was not statistically significant (6.16%, 9/146 vs 5.04%, 7/139; *P*=.88).

**Table 3.** Comparison of characteristics between high and low total citation per year study groups in CDM^a^-based pharmacoepidemiologic research through 2024.

Variable	Low total citation per year (N=139)	High total citation per year (N=146)	*P* value
Total citations per year, median (IQR)	1.00 (0.50-1.50)	4.00 (2.75-6.28)	<.001
Sample size, median (IQR)	168,046 (13,484-1,368,976)	376,677 (91,692-1,721,186)	.057
Number of centers, median (IQR)	6.00 (3.00-8.00)	7.00 (5.00-8.00)	.008
Data from the United States, n (%)	95 (68.35)	116 (79.45)	.045
Research exposure, n (%)			.003
Drug	71 (51.08)	50 (34.25)
Vaccine	68 (48.92%)	93 (63.70)
Medical device	0 (0.00)	3 (2.05)
Types of CDM n (%)			.24
OMOP^b^	36 (25.90)	30 (20.55)
VSD^c^	66 (47.48)	87 (59.59)
Sentinel or Mini-Sentinel	16 (11.51)	13 (8.90)
Others	21 (15.11)	16 (10.96)
Research directions, n (%)			.01
Safety	89 (64.03)	110 (75.34)
Effectiveness	9 (6.47)	5 (3.42)
Usage	32 (23.02)	15 (10.27)
Others	9 (6.47)	16 (10.96)
Including sensitive analysis, n (%)	36 (26.67)	50 (34.48)	.20
Including subgroup analysis n (%)	72 (52.17)	57 (39.04)	.04
Including reporting guideline n (%)	7 (5.04)	9 (6.16)	.88

a CDM: Common Data Model.

b OMOP: Observational Medical Outcomes Partnership.

c VSD: Vaccine Safety Datalink.

### Analysis of Authors and Institutions

Among the top 10 productive authors, Dr Nicola P Klein, director of the Vaccine Study Center at Kaiser Permanente Northern California, held the highest rank with 69 publications. Her work has an H-index of 29, and all publications were based on VSD. The second-ranked author, Dr Matthew F Daley, a senior clinical researcher at Kaiser Permanente Colorado, has 53 publications. The third position is held by Dr Allison L Naleway from Kaiser Permanente Northwest Center for Health Research, with a total of 48 publications. All of the top 10 authors are from the United States, with five affiliated with different Kaiser Permanente institutions. The publications by these 5 authors are all based on VSD (Multimedia Appendix 4).

The top 10 most productive institutions are listed in Multimedia Appendix 5, and the temporal trends of the top 5 are visualized in Figure 2C. Among these, eight institutions are based in the United States, and two are located in South Korea. Kaiser Permanente is the institution with the highest publication volume and the most rapid growth, with all publications based on VSD. South Korea’s Hallym University and Seoul National University began publishing around 2020, primarily utilizing OMOP and K-CDM in their studies.

### Analysis of Journals

The top 10 productive journals were listed in Table 4. Vaccine ranks first, with 49 publications, accounting for 16.0% (49/308) of the total publications in this field. Among these, studies were based on the following CDM: VSD (46/49, 93.90%), ConcePTION (2/49, 4.10%), and Sentinel (1/49, 2.00%). Pediatrics ranks second, with 24 publications, representing 7.79% (24/308) of the total. The CDMs used in these studies include VSD (21/24, 87.50%), PEDSnet (1/24, 4.17%), PEDSnet + OMOP (1/24, 4.17%), and PCORnet (1/24, 4.17%). *Pharmacoepidemiology and Drug Safety* published 22 studies, accounting for 7.20% (22/308) of the total publications. The CDM used in these studies included VSD (9/22, 40.91%), Sentinel (5/22, 22.73%), Mini-Sentinel (2/22, 9.09%), OMOP (2/22, 9.09%), Asian Pharmacoepidemiology Network (1/22, 4.55%), and newly created CDMs (3/22, 13.64%).

**Table 4.** Top 10 journals with most studies in pharmacoepidemiologic research based on CDMs^a^ through 2024.

Rank	Journal title	Country	Counts, n	IF^b^ (2023)	JCR^c^ (2023)	H-index	Total citations, n
1	*Vaccine*	United Kingdom	49	4.5	Q3	19	1207
2	*Pediatrics*	United States	24	6.2	Q2	18	1167
3	*Pharmacoepidemiology and Drug Safety*	United Kingdom	22	2.4	Q3	10	310
4	*Obstetrics and Gynecology*	United States	9	5.7	Q2	8	322
5	*Scientific Reports*	United Kingdom	8	3.8	Q3	4	38
6	*JAMA-Journal of the American Medical Association*	United States	7	63.1	Q1	7	925
7	*Jama Network Open*	United States	6	10.5	Q1	5	147
8	*American Journal of Epidemiology*	United States	6	5.0	Q2	4	187
9	*Drug Safety*	New Zealand	5	4.0	Q2	3	62
10	*Academic Pediatrics*	United States	4	3.0	Q3	4	34

a CDM: Common Data Model.

b IF: impact factor.

c JCR: journal citation reports.

As illustrated in Figure 2D, the number of pharmacoepidemiologic research studies based on CDMs in *Vaccine* shows notable variability, with marked increases in 2016 and 2022. In contrast, publication volume in *Pediatrics* and *JAMA Network Open* has remained relatively stable within this field.

### Analysis of Countries and Regions

The top 10 data source countries were listed in Table 5. An analysis of the 308 pharmacoepidemiology publications based on CDMs reveals that the leading countries in research output are the United States with 286 (92.86%) studies, South Korea with 53 (17.21%) studies, the United Kingdom with 11 (3.57%) studies.

**Table 5.** Top 10 data source countries in pharmacoepidemiologic research based on CDMs^a^ through 2024.

Rank	Country	Counts, n	Average citation counts per study	Average TCpY^b^ counts per study
1	United States	286	34.53	4.13
2	South Korea	53	9.26	2.15
3	United Kingdom	11	16.36	3.85
4	France	9	19.56	4.52
5	China	9	15.67	2.73
6	Spain	8	25.75	6.78
7	Germany	7	23.43	6.31
8	Japan	7	23.14	4.52
9	Netherlands	7	15.86	4.20
10	Denmark	6	10.83	1.86

a CDM: Common Data Model.

b TCpY: Total Citations per Year. Data from multiple countries were counted separately for each contributing country.

**Figure 3.**
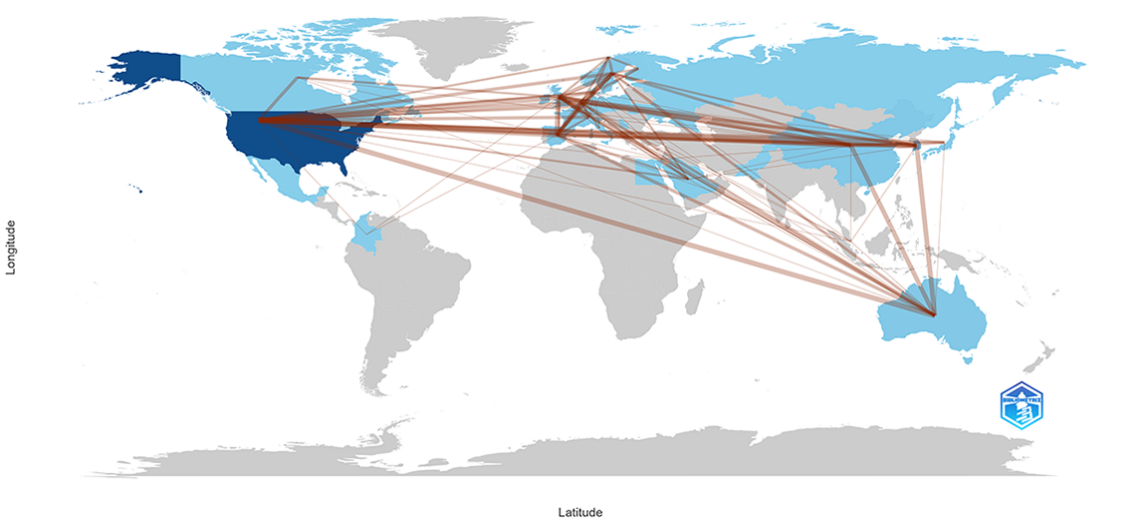
Global collaboration patterns among countries conducting CDM-based pharmacoepidemiologic research. CDM: Common Data Model; The thickness of the lines represents the strength of collaboration.

As illustrated in Figure 3, international collaboration is strong among the United States, Europe, Australia, and East Asia. The United States acts as a central hub for multinational collaborations, with substantial partnerships involving China, the United Kingdom, Spain, South Korea, and Australia. South Korea is also actively engaged in various international projects, maintaining particularly close connections with the United States and the United Kingdom.

### Analysis of Keywords

**Figure 4.**
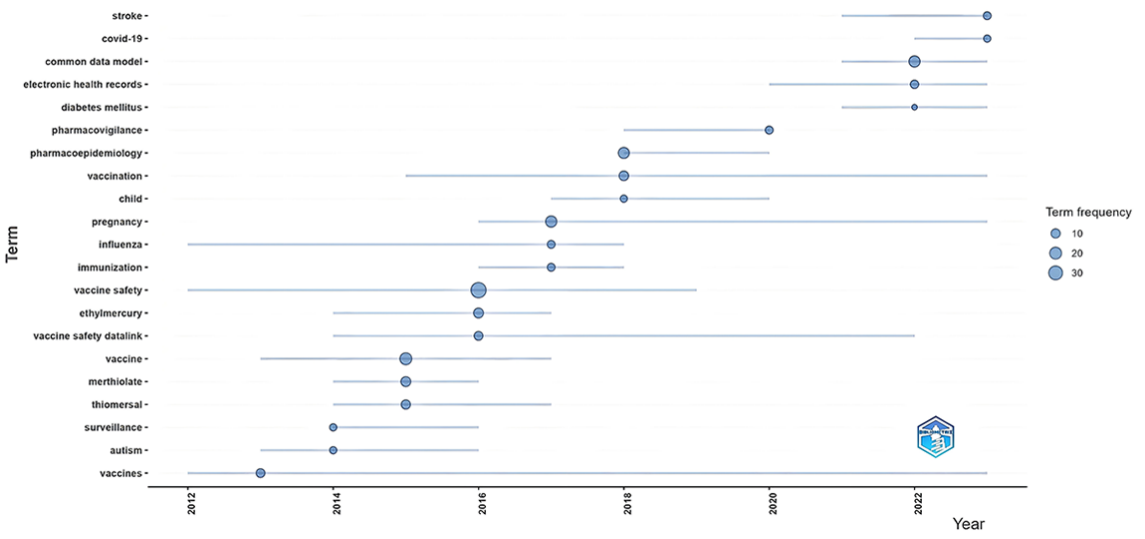
Trends in research topics over time in pharmacoepidemiologic research based on CDM through 2024. Dot: Represents the appearance of a specific topic or keyword. Dot size: Indicates the influence or research volume of the topic. Horizontal line: the duration or active period of the topic. CDM: Common Data Model.

The included publications encompass a total of 590 keywords. Figure 4 illustrates the temporal trends of popular research topics in CDMs based on pharmacoepidemiology. Since 2012, topics such as vaccine safety, immunization, and influenza have remained consistently active. Starting in 2020, research on COVID-19, stroke, and diabetes has increased significantly, with extensive use of electronic health record (EHR) data.

**Figure 5.**
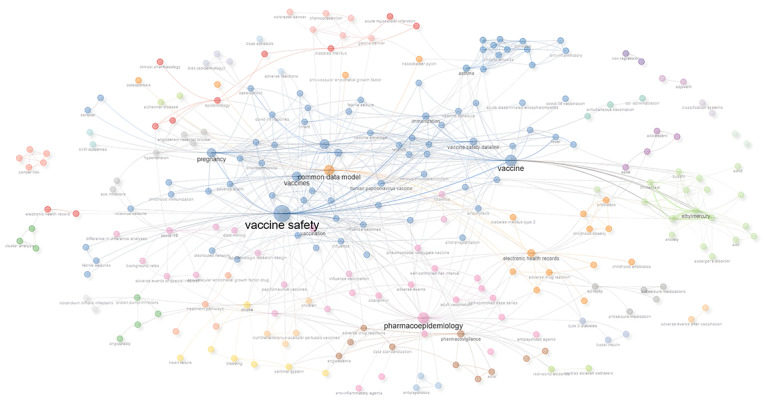
Network map of keywords in pharmacoepidemiologic research based on CDM. CDM: Common Data Model.

Figure 5 illustrates the trends in research topics. The nodes of the same color within a cluster represent closely related co-occurrences, with node size and link width varying based on the degree and strength of co-occurrence. “Vaccine safety” and “pharmacoepidemiology” occupy central positions in the network, showing strong connections with numerous surrounding topics. “Common data model” serves as another key topic, closely linked to “electronic health records” and “observational research.” The nodes in different colors indicate topic clusters, with the blue cluster primarily focusing on vaccine-related research, the orange cluster on drug monitoring and adverse reactions, and the brown cluster on drug regulation and risk assessment.

The dual-map overlay analysis illustrated the interdisciplinary citation structure of CDM-based pharmacoepidemiologic research. Most publications were clustered in the “Medicine, Medical, Clinical” domains and predominantly cited journals from the fields of “Health, Nursing, Medicine” and “Molecular Biology, Genetics,” suggesting strong disciplinary integration (Multimedia Appendix 6).

## Discussion

### Principal Findings

This study used bibliometric methods to analyze research trends in the application of CDMs in the field of pharmacoepidemiology, while also exploring research hot spots among highly cited studies in this field. The findings revealed that highly cited studies were predominantly originated from the United States, reflecting the central role of the United States researchers and institutions in this field, while South Korea’s influence has been rising rapidly in recent years. Included studies were often multicenter, interorganizational collaborations, leveraging extensive sample sizes and cross-regional data coverage to enhance the generalizability and scientific value of research outcomes, thereby increasing citation frequency [29]. Vaccine-related studies were a primary focus, highlighting the significance of the CDM in public health, particularly in supporting real-time assessment of drugs and vaccines and adverse event monitoring during global health crises by integrating data across regions and organizations [30]. Our findings provide essential guidance for researchers embarking on studies in this field, assisting in the identification of potential collaborators, influential studies, and exemplary work for reference, as well as in the selection of suitable journals for publication. It enhances the efficiency of establishing research networks and supports the effective dissemination and academic impact of new studies.

### Characteristics of Highly Cited Studies

The most cited publications globally have provided critical insights into adverse events associated with various vaccines, drug safety, and drug usage [14,30-33]. One particularly influential study, titled “Surveillance for Adverse Events After COVID-19 mRNA Vaccination,” monitored severe adverse events related to COVID-19 vaccines and has emerged as the most cited and impactful publication in this field [30]. This highlights the rapid response and proactive surveillance capabilities of CDM during public health emergencies, enabling timely evaluation of new vaccines.

We conducted a comparative analysis between globally highly cited studies and less cited ones. To mitigate the advantage of earlier publications due to absolute citation counts, we also grouped studies based on TCpY. The findings indicate that studies in the high-citation group tend to involve more institutions. Cross-institutional collaboration and data sharing provide significant practical guidance for research and policy-making. Multicenter studies, with larger participant numbers from diverse regions, enhance statistical power and ensure that findings are more generalizable for broader populations. Such studies allow researchers to capture a wider range of variables and cases, thus providing a robust data foundation for investigating rare diseases, uncommon adverse events, or smaller subpopulations [29,34,35].

Vaccine studies are more frequently highly cited compared to drug research, due to factors related to their public health impact and resource allocation. First, the high citation rates of vaccine research are closely tied to its critical role in public health. Global public health crises, such as the COVID-19 pandemic, have further elevated the priority of vaccine research, increasing its visibility and citation rates, as such studies are essential for policy-making and health interventions [36,37]. In addition, vaccine development often involves substantial public funding and data-sharing efforts, enabling wider access and greater citation of vaccine-related findings. In contrast, drug research is typically driven by pharmaceutical companies, with more restricted data availability and limited openness, which can constrain its citation impact [38].

### Key Contributors and Institutional Insights

The analysis of authors and institutions highlights the contributions of key researchers to the application of CDMs in pharmacoepidemiology. Core authors such as Nicola P Klein and Matthew F Daley demonstrate their extensive expertise and consistent productivity in this field. Among the top 10 most prolific authors, 5 are affiliated with different regions of Kaiser Permanente, a leading health care provider and nonprofit health plan in the United States since its establishment in 1945, currently serving members across 8 states and the District of Columbia [39]. In South Korea, Hallym University and Seoul National University have conducted multiple studies in this domain around 2020, using OMOP and K-CDM. Between 2016 and 2018, the Ministry of Food and Drug Safety and the Korea Institute of Drug Safety and Risk Management converted EHR of over 9 million patients into CDM, reflecting Korea’s progress in integrating and sharing cross-cohort data resources [40]. Subsequently, Korea developed the K-CDM specifically for pharmacovigilance systems, leveraging multi-center EHRs to monitor adverse drug reactions [41,42].

### Global Landscape and Collaborative Trends

The majority of studies in the high-citation group originated from the United States, which is the birthplace of several CDMs. As early as 2001, the United States established the VSD to track adverse events across multiple regions and populations [43]. Subsequently, the OMOP was released in 2007, the Sentinel was launched by the FDA in 2008, and the PCORnet initiative began in 2014, positioning the United States at the forefront of CDM development and usage worldwide [43]. Moreover, the United States excels in drug and vaccine surveillance through the VSD and Sentinel systems. VSD provides real-time updates and comprehensive vaccine data, while Sentinel builds on VSD with tools enabling direct, standardized analyses, streamlining research processes [11].

In addition, the United States is the primary contributor in terms of publication volume and frequency, followed by South Korea. These countries have made significant contributions to advancing knowledge in this field. Notably, co-occurrence analysis highlights active participation from other countries in collaborative networks, with strong connections observed among North America, Europe, and East Asia. However, we observed that most international collaborations occur primarily among limited-income countries, with limited participation from other nations, particularly in limited-income countries. Given that CDMs are designed to harmonize data from multiple databases and facilitate standardized analyses [44], future research should focus on fostering broader and more impactful international collaborations from countries at different levels of development. Furthermore, the analysis of institutional productivity provides practical insights that may directly benefit researchers seeking potential collaborators. Our results highlight that Kaiser Permanente and CDC in the United States, both major users of VSD, represent key contributors to CDM-based vaccine research in the United States. Meanwhile, institutions such as Hallym University and Seoul National University in South Korea have emerged as important centers of CDM implementation, primarily using OMOP and K-CDM. Recognizing these leading institutions and their methodological preferences may facilitate strategic planning of future collaborative studies, enabling researchers to align their research agendas, optimize resource allocation, and ultimately enhance the global impact of CDM-based pharmacoepidemiologic research.

### Implications for Future Research and Practice

To foster more equitable global participation, particularly from low- and middle-income countries, future efforts should prioritize improving access to CDM infrastructure. This includes the provision of open-source tools, standardized implementation workflows, and multilingual technical documentation. In parallel, the establishment of dedicated funding mechanisms and long-term capacity-building programs—such as technical training and data transformation support—will be essential to enable meaningful international collaboration. These strategies aim to bridge existing disparities and promote a more inclusive and diverse global CDM research ecosystem.

Furthermore, the findings of this study offer practical implications for clinical decision-making and real-world health policy. By identifying highly cited, multicenter studies—many of which focus on vaccine safety and effectiveness—this analysis highlights how CDM-based research can rapidly generate actionable evidence during public health emergencies. The global collaborative networks and methodological practices identified in this study may serve as a reference for designing future pharmacoepidemiologic studies that directly inform clinical guidelines, pharmacovigilance strategies, and regulatory decision-making. Strengthening the application of CDMs in such contexts can ultimately improve patient safety, treatment optimization, and health system responsiveness.

### Strengths and Limitations

This study has some strengths. To our knowledge, this bibliometric study is the first of its kind in the field of application of CDMs in pharmacoepidemiology, to summarize the publication characteristics. Using the systematic review, we screened studies that met the inclusion criteria, ensuring the reliability of the selected studies. Furthermore, we imposed no restrictions on publication date or on the types of CDMs, which allowed us to include studies globally. This approach enables comparisons across different periods, countries, and types of CDMs. It must be acknowledged that our research has several modest limitations. First, the most significant limitation of our study lies in the inherent biases of citation analysis. These include the tendency for earlier publications to accumulate higher citation counts, journal and author self-citations, incomplete citation data, and omissions, all of which may affect citation rates [23,45,46]. To mitigate such biases, we attempted to use TCpY as an alternative metric. Second, we selected WoSCC based on previous studies [18,36], as it allows standardized citation-based analysis. However, WoSCC does not index all eligible studies. Among the 308 included studies, 285 were found in WoSCC and therefore included in citation-related analyses. The remaining 23 studies lacked citation data and were excluded from this component. To evaluate whether their exclusion introduced selection bias, we compared key characteristics between the 2 groups. As shown in Multimedia Appendix 3, no statistically significant differences were found. Therefore, the potential bias introduced by excluding non-WoSCC studies is likely minimal. Third, we included only English and Chinese studies, which means that studies published in other languages were excluded. This may lead to the omission of some research. However, we searched the Virtual Health Library to ensure that literature published in Latin America was also covered to the greatest extent possible. Fourth, the use of the bibliometrix R package, while powerful for large-scale bibliometric analysis, may introduce methodological limitations. For example, its analytical outputs rely on the quality and structure of the input data and specific co-occurrence or clustering algorithms [26]. To minimize these effects, we carefully cleaned and standardized the dataset and interpreted the results in combination with manual validation and descriptive statistics. In addition, the release dates of different CDMs vary, with earlier CDMs having accumulated a higher publication volume and citations compared to those released more recently.

### Conclusions

This bibliometric analysis provides a comprehensive overview of the application of CDMs in pharmacoepidemiology. The findings indicate a significant increase in publications over time, with the United States leading in both publication volume and citation counts. Notably, high-citation studies often involve multicenter studies, particularly focusing on vaccine, underscoring the importance of collaborative research efforts. These insights emphasize the critical role of CDMs in facilitating large-scale, collaborative pharmacoepidemiologic research, as well as provide researchers with insights for selecting collaborators and choosing journals for publication. To enhance global equity and clinical relevance, further efforts should support infrastructure accessibility, international cooperation, and the integration of CDM-based evidence into public health and regulatory decision-making.

## Supplementary material

10.2196/72225 Multimedia Appendix 1 Search strategies.

10.2196/72225 Multimedia Appendix 2 Top 10 most cited articles in pharmacoepidemiologic research based on Common Data Models through 2024.

10.2196/72225 Multimedia Appendix 3 Comparison of study characteristics between articles included and those not included in the Web of Science Core Collection.

10.2196/72225 Multimedia Appendix 4 Top 10 authors with most articles in pharmacoepidemiologic research based on Common Data Models through 2024.

10.2196/72225 Multimedia Appendix 5 Top 10 institutions with most articles in pharmacoepidemiologic research based on Common Data Models through 2024.

10.2196/72225 Multimedia Appendix 6 Dual-map overlay of journal citation trajectories in Common Data Model–based pharmacoepidemiologic research.

10.2196/72225 Checklist 1 PRISMA (Preferred Reporting Items for Systematic Reviews and Meta-Analyses) 2020 checklist.

## Abbreviations

AsPEN: Asian Pharmacoepidemiology Network
CDM: Common Data Model
EHR: electronic health record
OMOP: Observational Medical Outcomes Partnership
PRISMA: Preferred Reporting Items for Systematic Reviews and Meta-Analyses
TCpY: Total Citations per Year
VSD: Vaccine Safety Datalink
WoSCC: Web of Science Core Collection
