# Supplementary material for: Pharmacoepidemiologic Research Based on Common Data Models: Systematic Review and Bibliometric Analysis
Source: JMIR Med Inform. 2025 Jul 28;13:e72225. doi: 10.2196/72225 (PMC12303556; doi:10.2196/72225)
Supplement: Multimedia Appendix 1 [file medinform-v13-e72225-s001.docx]

**Appendix 1: Search strategies (Search deadline: January 22, 2024)**

1. ***Search strategy for PubMed***

"Common data model"[Title/Abstract] OR "Observational Health Data Sciences and Informatics"[Title/Abstract] OR "OHDSI"[Title/Abstract] OR "Observational Medical Outcomes Partnership"[Title/Abstract] OR "OMOP"[Title/Abstract] OR "Clinical Data Interchange Standards Consortium"[Title/Abstract] OR "CDISC"[Title/Abstract] OR "Study Data Tabulation Model"[Title/Abstract] OR "SDTM"[Title/Abstract] OR "Clinical Data Acquisition Standards Harmonization"[Title/Abstract] OR "CDASH"[Title/Abstract] OR "Patient-Centered Clinical Research Network"[Title/Abstract] OR "PCORnet"[Title/Abstract] OR "Patient-Centered Outcomes Research Institute"[Title/Abstract] OR "PCORI"[Title/Abstract] OR "Health Level Seven"[Title/Abstract] OR "HL7"[Title/Abstract] OR "HL-7"[Title/Abstract] OR "Fast Healthcare Interoperability Resources"[Title/Abstract] OR "FHIR"[Title/Abstract] OR ("sentinel"[Title/Abstract] AND "data model"[Title/Abstract]) OR "sentinel system*"[Title/Abstract] OR "sentinel initiative"[Title/Abstract] OR "SCDM"[Title/Abstract] OR "Mini-Sentinel"[Title/Abstract] OR "MSCDM"[Title/Abstract] OR "i2b2"[Title/Abstract] OR "informatics for integrating biology the bedside"[Title/Abstract] OR "Informatics for Integrating Biology and the Bedside"[Title/Abstract] OR "ConcePTION project"[Title/Abstract] OR "NorPreSS"[Title/Abstract] OR "Nordic Pregnancy Drug Safety Studies "[Title/Abstract] OR "Vaccine Safety Datalink"[Title/Abstract] OR ("VSD"[Title/Abstract] AND "vaccin*"[Title/Abstract]) OR "Pediatric Learning Health System"[Title/Abstract] OR "PEDSnet"[Title/Abstract] OR "CRN-VDW"[Title/Abstract] OR "Virtual Data Warehouse"[Title/Abstract] OR "Cancer Research Network"[Title/Abstract] OR "Health Care Systems Research Network"[Title/Abstract] OR "HCSRN"[Title/Abstract] OR "CESR"[Title/Abstract] OR "HMORN"[Title/Abstract] OR "Health Maintenance Organization Research Network"[Title/Abstract] OR "VAESCO"[Title/Abstract] OR "Asian Pharmacoepidemiology Network"[Title/Abstract] OR " Asian Pharmacoepidemiology Network"[Title/Abstract]

1. ***Search strategy for WOS***

((((((((((((((((((((((((((((((((((((((((((((((TI=("Common data model")) OR TI=("Observational Health Data Sciences and Informatics")) OR TI=(OHDSI)) OR TI=("Observational Medical Outcomes Partnership")) OR TI=(OMOP)) OR TI=("Clinical Data Interchange Standards Consortium")) OR TI=(CDISC)) OR TI=("Study Data Tabulation Model")) OR TI=(SDTM)) OR TI=("Clinical Data Acquisition Standards Harmonization")) OR TI=(CDASH)) OR TI=("Patient-Centered Clinical Research Network")) OR TI=(PCORnet)) OR TI=("Patient-Centered Outcomes Research Institute")) OR TI=(PCORI)) OR TI=("Health Level Seven")) OR TI=(HL7)) OR TI=(HL-7)) OR TI=("Fast Healthcare Interoperability Resources")) OR TI=(FHIR)) OR (TI=(sentinel) AND TI=("data model"))) OR TI=("sentinel system*")) OR TI=("sentinel initiative")) OR TI=(SCDM)) OR TI=(Mini-Sentinel)) OR TI=(MSCDM)) OR TI=(i2b2)) OR TI=("Informatics for integrating Biology & the Bedside")) OR TI=("Informatics for Integrating Biology and the Bedside")) OR TI=("ConcePTION project")) OR TI=(NorPreSS)) OR TI=("Nordic Pregnancy Drug Safety Studies")) OR TI=("Vaccine Safety Datalink")) OR (TI=(VSD) AND TI=("vaccin*"))) OR TI=("Pediatric Learning Health System")) OR TI=(PEDSnet)) OR TI=(CRN-VDW)) OR TI=("Virtual Data Warehouse")) OR TI=("Cancer Research Network")) OR TI=(" Health Care Systems Research Network")) OR TI=(HCSRN)) OR TI=(CESR)) OR TI=("Center for Effectiveness and Safety Research")) OR TI=(HMORN)) OR TI=("Health Maintenance Organization Research Network")) OR TI=(VAESCO)) OR TI=("Vaccine Adverse Events Surveillance and Communication")) OR TI=(" Asian Pharmacoepidemiology Network ") OR (((((((((((((((((((((((((((((((((((((((((((((((AB=("Common data model")) OR AB=("Observational Health Data Sciences and Informatics")) OR AB=(OHDSI)) OR AB=("Observational Medical Outcomes Partnership")) OR AB=(OMOP)) OR AB=("Clinical Data Interchange Standards Consortium")) OR AB=(CDISC)) OR AB=("Study Data Tabulation Model")) OR AB=(SDTM)) OR AB=("Clinical Data Acquisition Standards Harmonization")) OR AB=(CDASH)) OR AB=("Patient-Centered Clinical Research Network")) OR AB=(PCORnet)) OR AB=("Patient-Centered Outcomes Research Institute")) OR AB=(PCORI)) OR AB=("Health Level Seven")) OR AB=(HL7)) OR AB=(HL-7)) OR AB=("Fast Healthcare Interoperability Resources")) OR AB=(FHIR)) OR (AB=(sentinel) AND AB=("data model"))) OR AB=("sentinel system*")) OR AB=("sentinel initiative")) OR AB=(SCDM)) OR AB=(Mini-Sentinel)) OR AB=(MSCDM)) OR AB=(i2b2)) OR AB=("Informatics for integrating Biology & the Bedside")) OR AB=("Informatics for Integrating Biology and the Bedside")) OR AB=("ConcePTION project")) OR AB=(NorPreSS)) OR AB=("Nordic Pregnancy Drug Safety Studies")) OR AB=("Vaccine Safety Datalink")) OR (AB=(VSD) AND AB=("vaccin*"))) OR AB=("Pediatric Learning Health System")) OR AB=(PEDSnet)) OR AB=(CRN-VDW)) OR AB=("Virtual Data Warehouse")) OR AB=("Cancer Research Network")) OR AB=(" Health Care Systems Research Network")) OR AB=(HCSRN)) OR AB=(CESR)) OR AB=("Center for Effectiveness and Safety Research")) OR AB=(HMORN)) OR AB=("Health Maintenance Organization Research Network")) OR AB=(VAESCO)) OR AB=("Vaccine Adverse Events Surveillance and Communication")) OR AB=(" Asian Pharmacoepidemiology Network ")

1. ***Search strategy for Embase***

'common data model':ti,ab OR 'observational health data sciences and informatics':ti,ab OR 'ohdsi':ti,ab OR 'observational medical outcomes partnership':ti,ab OR 'omop':ti,ab OR 'clinical data interchange standards consortium':ti,ab OR 'cdisc':ti,ab OR 'study data tabulation model':ti,ab OR 'sdtm':ti,ab OR 'clinical data acquisition standards harmonization':ti,ab OR 'cdash':ti,ab OR 'patient-centered clinical research network':ti,ab OR 'pcornet':ti,ab OR 'patient-centered outcomes research institute':ti,ab OR 'pcori':ti,ab OR 'health level seven':ti,ab OR 'hl7':ti,ab OR 'hl-7':ti,ab OR 'fast healthcare interoperability resources':ti,ab OR 'fhir':ti,ab OR ('sentinel':ti,ab AND 'data model':ti,ab) OR 'sentinel system*':ti,ab OR 'sentinel initiative':ti,ab OR 'scdm':ti,ab OR 'mini-sentinel':ti,ab OR 'mscdm':ti,ab OR 'i2b2':ti,ab OR 'informatics for integrating biology & the bedside':ti,ab OR 'informatics for integrating biology and the bedside':ti,ab OR 'conception project':ti,ab OR 'norpress':ti,ab OR 'nodic pregnancy drug safety studies':ti,ab OR 'vaccine safety datalink':ti,ab OR ('vsd':ti,ab AND 'vaccin*':ti,ab) OR 'pediatric learning health system':ti,ab OR 'pedsnet':ti,ab OR 'crn-vdw':ti,ab OR 'virtual data warehouse':ti,ab OR 'cancer research network':ti,ab OR 'health care systems research network':ti,ab OR 'hcsrn':ti,ab OR 'cesr':ti,ab OR 'center for effectiveness and safety network':ti,ab OR 'hmorn':ti,ab OR 'health maintenance organization research network':ti,ab OR 'vaesco':ti,ab OR 'vaccine adverse events surveillance and communication':ti,ab OR 'asian pharmacoepidemiology network':ti,ab

1. ***Search strategy for Scopus***

TITLE-ABS("Common data model") OR TITLE-ABS("Observational Health Data Sciences and Informatics") OR TITLE-ABS(OHDSI) OR TITLE-ABS("Observational Medical Outcomes Partnership") OR TITLE-ABS(OMOP) OR TITLE-ABS("Clinical Data Interchange Standards Consortium") OR TITLE-ABS(CDISC) OR TITLE-ABS("Study Data Tabulation Model") OR TITLE-ABS(SDTM) OR TITLE-ABS("Clinical Data Acquisition Standards Harmonization") OR TITLE-ABS(CDASH) OR TITLE-ABS("Patient-Centered Clinical Research Network") OR TITLE-ABS(PCORnet) OR TITLE-ABS("Patient-Centered Outcomes Research Institute") OR TITLE-ABS(PCORI) OR TITLE-ABS("Health Level Seven") OR TITLE-ABS(HL7) OR TITLE-ABS(HL-7) OR TITLE-ABS("Fast Healthcare Interoperability Resources") OR TITLE-ABS(FHIR) OR TITLE-ABS ((sentinel) AND ("data model")) OR TITLE-ABS("sentinel system*") OR TITLE-ABS ("sentinel initiative") OR TITLE-ABS (SCDM) OR TITLE-ABS(Mini-Sentinel) OR TITLE-ABS(MSCDM) OR TITLE-ABS(i2b2) OR TITLE-ABS("Informatics for integrating Biology & the Bedside") OR TITLE-ABS("Informatics for Integrating Biology and the Bedside") OR TITLE-ABS("ConcePTION project") OR TITLE-ABS(NorPreSS) OR TITLE-ABS("Nordic Pregnancy Drug Safety Studies") OR TITLE-ABS("Vaccine Safety Datalink") OR TITLE-ABS ((VSD) AND (vaccin*)) OR TITLE-ABS("Pediatric Learning Health System") OR TITLE-ABS(PEDSnet) OR TITLE-ABS(CRN-VDW) OR TITLE-ABS("Virtual Data Warehouse") OR TITLE-ABS("Cancer Research Network") OR TITLE-ABS("Health Care Systems Research Network") OR TITLE-ABS(HCSRN) OR TITLE-ABS(CESR) OR TITLE-ABS("Center for Effectiveness and Safety Research") OR TITLE-ABS(HMORN) OR TITLE-ABS("Health Maintenance Organization Research Network") OR TITLE-ABS(VAESCO) OR TITLE-ABS("Vaccine Adverse Events Surveillance and Communication") OR TITLE-ABS("Asian Pharmacoepidemiology Network")

1. ***Search strategy for VHL***

("Common data model") OR ("Observational Health Data Sciences and Informatics") OR (“OHDSI”) OR ("Observational Medical Outcomes Partnership") OR ("OMOP") OR ("Clinical Data Interchange Standards Consortium") OR ("CDISC") OR ("Study Data Tabulation Model") OR ("SDTM") OR ("Clinical Data Acquisition Standards Harmonization") OR ("CDASH") OR ("Patient-Centered Clinical Research Network") OR ("PCORnet") OR ("Patient-Centered Outcomes Research Institute") OR ("PCORI") OR ("Health Level Seven") OR ("HL7") OR ("HL-7") OR ("Fast Healthcare Interoperability Resources") OR ("FHIR") OR (("sentinel") AND ("data model")) OR ("sentinel system*") OR ("sentinel initiative") OR ("SCDM") OR ("Mini-Sentinel") OR ("MSCDM") OR ("i2b2") OR ("Informatics for integrating Biology & the Bedside") OR ("Informatics for Integrating Biology and the Bedside") OR ("ConcePTION project") OR ("NorPreSS") OR ("Nordic Pregnancy Drug Safety Studies") OR ("Vaccine Safety Datalink") OR (("VSD") AND ("vaccin*")) OR ("Pediatric Learning Health System") OR ("PEDSnet") OR ("CRN-VDW") OR ("Virtual Data Warehouse") OR ("Cancer Research Network") OR ("Health Care Systems Research Network") OR ("HCSRN") OR ("CESR") OR ("Center for Effectiveness and Safety Research") OR ("HMORN") OR ("Health Maintenance Organization Research Network") OR ("VAESCO") OR ("Vaccine Adverse Events Surveillance and Communication") OR ("Asian Pharmacoepidemiology Network")

1. ***Search strategy for* *CNKI***

TI="通用数据模型" OR TI="OMOP" OR TI="观测性医疗结果合作组织" OR TI="观察医疗结果合作项目" OR TI="观察医疗结果合作伙伴关系" OR TI="观察性医学产出合作项目" OR TI="OHDSI" OR TI="观察健康数据科学和信息学" OR TI="观察性健康数据科学和信息学" OR TI="健康观测数据科学和信息学" OR TI="观察性健康医疗数据科学与信息学" OR TI="CDISC" OR TI="临床数据交换标准协会" OR TI="SDTM" OR TI="研究数据制表模型" OR TI="研究数据表格模型" OR TI="研究数据列表模型" OR TI="研究数据制表标准" OR TI="CDASH" OR TI="临床数据获取协调标准" OR TI="临床数据采集标准" OR TI="临床数据获取标准协调" OR TI="临床数据获取的标准协调" OR TI="PCORnet" OR TI="以患者为中心的国家级临床研究网络" OR TI="国家患者中心临床研究网络" OR TI="患者为中心的临床研究网络" OR TI="PCORI" OR TI="患者导向医疗效果研究所" OR TI="以患者为中心结局研究机构" OR TI="患者导向医疗质量研究所" OR TI="以患者为中心的临床结局研究中心" OR TI="HL7" OR TI="美国卫生信息传输标准" OR TI="FHIR" OR TI="快速医疗互操作资源" OR TI="快速医疗互操作性资源" OR TI="SCDM" OR TI="哨点通用数据模型" OR TI="Mini-Sentinel" OR TI="迷你哨点" OR TI="微哨点" OR TI="Vaccine Safety Datalink" OR TI="疫苗安全性数据库链接" OR TI="疫苗安全数据库网" OR TI="疫苗安全数据系统" OR TI="疫苗安全数据链" OR TI="疫苗安全性数据链" OR TI="疫苗安全性数据系统" OR TI="PEDSnet" OR TI="儿科学习型健康医疗系统" OR TI="儿科学习健康系统" OR TI="VAESCO" OR TI="疫苗不良事件监测和沟通" OR TI="疫苗不良事件监测沟通" OR TI="疫苗不良事件监测与通信" OR TI="疫苗不良事件监测和通信" OR TI="Asian Pharmacoepidemiology Network" OR TI="亚洲药物流行病学网络" OR TI="亚洲药物流行病学合作网" OR TI="亚洲药物流行病学协作网" OR TI="i2b2" OR TI="ConcePTION通用数据模型" OR TI="NorPreSS" OR TI="北欧妊娠药物安全性研究" OR TI="CRN-VDW" OR TI="癌症研究网络虚拟数据仓库" OR TI="HCSRN" OR TI="卫生保健系统研究网络" OR TI="CESR" OR TI="有效性和安全性研究中心" OR TI="HMORN" OR TI="健康维护组织研究网络" OR AB="通用数据模型" OR AB="OMOP" OR AB="观测性医疗结果合作组织" OR AB="观察医疗结果合作项目" OR AB="观察医疗结果合作伙伴关系" OR AB="观察性医学产出合作项目" OR AB="OHDSI" OR AB="观察健康数据科学和信息学" OR AB="观察性健康数据科学和信息学" OR AB="健康观测数据科学和信息学" OR AB="观察性健康医疗数据科学与信息学" OR AB="CDISC" OR AB="临床数据交换标准协会" OR AB="SDTM" OR AB="研究数据制表模型" OR AB="研究数据表格模型" OR AB="研究数据列表模型" OR AB="研究数据制表标准" OR AB="CDASH" OR AB="临床数据获取协调标准" OR AB="临床数据采集标准" OR AB="临床数据获取标准协调" OR AB="临床数据获取的标准协调" OR AB="PCORnet" OR AB="以患者为中心的国家级临床研究网络" OR AB="国家患者中心临床研究网络" OR AB="患者为中心的临床研究网络" OR AB="PCORI" OR AB="患者导向医疗效果研究所" OR AB="以患者为中心结局研究机构" OR AB="患者导向医疗质量研究所" OR AB="以患者为中心的临床结局研究中心" OR AB="HL7" OR AB="美国卫生信息传输标准" OR AB="FHIR" OR AB="快速医疗互操作资源" OR AB="快速医疗互操作性资源" OR AB="SCDM" OR AB="哨点通用数据模型" OR AB="Mini-Sentinel" OR AB="迷你哨点" OR AB="微哨点" OR AB="Vaccine Safety Datalink" OR AB="疫苗安全性数据库链接" OR AB="疫苗安全数据库网" OR AB="疫苗安全数据系统" OR AB="疫苗安全数据链" OR AB="疫苗安全性数据链" OR AB="疫苗安全性数据系统" OR AB="PEDSnet" OR AB="儿科学习型健康医疗系统" OR AB="儿科学习健康系统" OR AB="VAESCO" OR AB="疫苗不良事件监测和沟通" OR AB="疫苗不良事件监测沟通" OR AB="疫苗不良事件监测与通信" OR AB="疫苗不良事件监测和通信" OR AB="Asian Pharmacoepidemiology Network" OR AB="亚洲药物流行病学网络" OR AB="亚洲药物流行病学合作网" OR AB="亚洲药物流行病学协作网" OR AB="i2b2" OR AB="ConcePTION通用数据模型" OR AB="NorPreSS" OR AB="北欧妊娠药物安全性研究" OR AB="CRN-VDW" OR AB="癌症研究网络虚拟数据仓库" OR AB="HCSRN" OR AB="卫生保健系统研究网络" OR AB="CESR" OR AB="有效性和安全性研究中心" OR AB="HMORN" OR AB="健康维护组织研究网络"

1. ***Search strategy for* *Wanfang data***

题名：("通用数据模型" ) OR 摘要：("通用数据模型") OR 题名：("OMOP" ) OR 摘要：("OMOP ") OR 题名：("观测性医疗结果合作组织" ) OR 摘要：("观测性医疗结果合作组织") OR 题名：("观察医疗结果合作项目" ) OR 摘要：("观察医疗结果合作项目") OR 题名：("观察医疗结果合作伙伴关系" ) OR 摘要：("观察医疗结果合作伙伴关系") OR 题名：("观察性医学产出合作项目" ) OR 摘要：("观察性医学产出合作项目") OR 题名：("OHDSI" ) OR 摘要：("OHDSI ") OR 题名：("观察健康数据科学和信息学" ) OR 摘要：("观察健康数据科学和信息学") OR 题名：("观察性健康数据科学和信息学" ) OR 摘要：("观察性健康数据科学和信息学") OR 题名：("健康观测数据科学和信息学" ) OR 摘要：("健康观测数据科学和信息学") OR 题名：("观察性健康医疗数据科学与信息学" ) OR 摘要：("观察性健康医疗数据科学与信息学") OR 题名：("CDISC" ) OR 摘要：("CDISC ") OR 题名：("临床数据交换标准协会" ) OR 摘要：("临床数据交换标准协会") OR 题名：("SDTM" ) OR 摘要：("SDTM ") OR 题名：("研究数据制表模型" ) OR 摘要：("研究数据制表模型") OR 题名：("研究数据表格模型" ) OR 摘要：("研究数据表格模型") OR 题名：("研究数据列表模型" ) OR 摘要：("研究数据列表模型") OR 题名：("研究数据制表标准" ) OR 摘要：("研究数据制表标准") OR 题名：("CDASH" ) OR 摘要：("CDASH ") OR 题名：("临床数据获取协调标准" ) OR 摘要：("临床数据获取协调标准") OR 题名：("临床数据采集标准" ) OR 摘要：("临床数据采集标准") OR 题名：("临床数据获取标准协调" ) OR 摘要：("临床数据获取标准协调") OR 题名：("临床数据获取的标准协调" ) OR 摘要：("临床数据获取的标准协调") OR 题名：("PCORnet" ) OR 摘要：("PCORnet ") OR 题名：("以患者为中心的国家级临床研究网络" ) OR 摘要：("以患者为中心的国家级临床研究网络") OR 题名：("国家患者中心临床研究网络" ) OR 摘要：("国家患者中心临床研究网络") OR 题名：("患者为中心的临床研究网络" ) OR 摘要：("患者为中心的临床研究网络") OR 题名：("PCORI" ) OR 摘要：("PCORI ") OR 题名：("患者导向医疗效果研究所" ) OR 摘要：("患者导向医疗效果研究所") OR 题名：("以患者为中心结局研究机构" ) OR 摘要：("以患者为中心结局研究机构") OR 题名：("患者导向医疗质量研究所" ) OR 摘要：("患者导向医疗质量研究所") OR 题名：("以患者为中心的临床结局研究中心" ) OR 摘要：("以患者为中心的临床结局研究中心") OR 题名：("HL7" ) OR 摘要：("HL7") OR 题名：("美国卫生信息传输标准" ) OR 摘要：("美国卫生信息传输标准") OR 题名：("FHIR" ) OR 摘要：("FHIR ") OR 题名：("快速医疗互操作资源" ) OR 摘要：("快速医疗互操作资源") OR 题名：("快速医疗互操作性资源" ) OR 摘要：("快速医疗互操作性资源") OR 题名：("SCDM" ) OR 摘要：("SCDM ") OR 题名：("哨点通用数据模型" ) OR 摘要：("哨点通用数据模型") OR 题名：("Mini-Sentinel" ) OR 摘要：("Mini-Sentinel ") OR 题名：("迷你哨点" ) OR 摘要：("迷你哨点") OR 题名：("微哨点" ) OR 摘要：("微哨点") OR 题名：("Vaccine Safety Datalink" ) OR 摘要：("Vaccine Safety Datalink ") OR 题名：("疫苗安全性数据库链接" ) OR 摘要：("疫苗安全性数据库链接") OR 题名：("疫苗安全数据库网" ) OR 摘要：("疫苗安全数据库网") OR 题名：("疫苗安全数据系统" ) OR 摘要：("疫苗安全数据系统") OR 题名：("疫苗安全数据链" ) OR 摘要：("疫苗安全数据链") OR 题名：("疫苗安全性数据链" ) OR 摘要：("疫苗安全性数据链") OR 题名：("疫苗安全性数据系统" ) OR 摘要：("疫苗安全性数据系统") OR 题名：("PEDSnet" ) OR 摘要：("PEDSnet ") OR 题名：("儿科学习型健康医疗系统" ) OR 摘要：("儿科学习型健康医疗系统") OR 题名：("儿科学习健康系统" ) OR 摘要：("儿科学习健康系统") OR 题名：("VAESCO" ) OR 摘要：("VAESCO ") OR 题名：("疫苗不良事件监测和沟通" ) OR 摘要：("疫苗不良事件监测和沟通") OR 题名：("疫苗不良事件监测沟通" ) OR 摘要：("疫苗不良事件监测沟通") OR 题名：("疫苗不良事件监测与通信" ) OR 摘要：("疫苗不良事件监测与通信") OR 题名：("疫苗不良事件监测和通信" ) OR 摘要：("疫苗不良事件监测和通信") OR 题名：("Asian Pharmacoepidemiology Network" ) OR 摘要：("Asian Pharmacoepidemiology Network") OR 题名：("亚洲药物流行病学网络" ) OR 摘要：("亚洲药物流行病学网络") OR 题名：("亚洲药物流行病学合作网" ) OR 摘要：("亚洲药物流行病学合作网") OR 题名：("亚洲药物流行病学协作网" ) OR 摘要：("亚洲药物流行病学协作网") OR 题名：("i2b2" ) OR 摘要：("i2b2") OR 题名：(" ConcePTION通用数据模型" ) OR 摘要：("ConcePTION通用数据模型") OR 题名：("NORPreSS" ) OR 摘要：("NORPreSS ") OR 题名：("北欧妊娠药物安全性研究" ) OR 摘要：("北欧妊娠药物安全性研究") OR 题名：("CRN-VDW" ) OR 摘要：("CRN-VDW ") OR 题名：("癌症研究网络虚拟数据仓库" ) OR 摘要：("癌症研究网络虚拟数据仓库") OR 题名：("HCSRN" ) OR 摘要：("HCSRN ") OR 题名：("卫生保健系统研究网络" ) OR 摘要：("卫生保健系统研究网络") OR 题名：("CESR" ) OR 摘要：("CESR ") OR 题名：("有效性和安全性研究中心" ) OR 摘要：("有效性和安全性研究中心") OR 题名：("HMORN" ) OR 摘要：("HMORN ") OR 题名：("健康维护组织研究网络") OR 摘要：("健康维护组织研究网络")

1. ***Search strategy for VIP***

T="OMOP" OR T="OHDSI" OR T="CDISC" OR T="SDTM" OR T="CDASH" OR T="PCORnet" OR T="PCORI" OR T="HL7" OR T="FHIR" OR T="SCDM" OR T="Mini-Sentinel" OR T="PEDSnet" OR T="VAESCO" OR T="i2b2" OR T="NorPreSS" OR T="CRN-VDW" OR T="HCSRN" OR T="CESR" OR T="HMORN" OR R="OMOP" OR R="OHDSI" OR R="CDISC" OR R="SDTM" OR R="CDASH" OR R="PCORnet" OR R="PCORI" OR R="HL7" OR R="FHIR" OR R="SCDM" OR R="Mini-Sentinel" OR R="PEDSnet" OR R="VAESCO" OR R="i2b2" OR R="NorPreSS" OR R="CRN-VDW" OR R="HCSRN" OR R="CESR" OR R="HMORN"

1. ***Search strategy for Sinomed***

"通用数据模型" [摘要:智能] OR "OMOP" [摘要:智能] OR "观测性医疗结果合作组织" [摘要:智能] OR "观察医疗结果合作项目" [摘要:智能] OR "观察医疗结果合作伙伴关系" [摘要:智能] OR "观察性医学产出合作项目" [摘要:智能] OR "OHDSI" [摘要:智能] OR "观察健康数据科学和信息学" [摘要:智能] OR "观察性健康数据科学和信息学" [摘要:智能] OR "健康观测数据科学和信息学" [摘要:智能] OR "观察性健康医疗数据科学与信息学" [摘要:智能] OR "CDISC" [摘要:智能] OR "临床数据交换标准协会" [摘要:智能] OR "SDTM" [摘要:智能] OR "研究数据制表模型" [摘要:智能] OR "研究数据表格模型" [摘要:智能] OR "研究数据列表模型" [摘要:智能] OR "研究数据制表标准" [摘要:智能] OR "CDASH" [摘要:智能] OR "临床数据获取协调标准" [摘要:智能] OR "临床数据采集标准" [摘要:智能] OR "临床数据获取标准协调" [摘要:智能] OR "临床数据获取的标准协调" [摘要:智能] OR "PCORnet" [摘要:智能] OR "以患者为中心的国家级临床研究网络" [摘要:智能] OR "国家患者中心临床研究网络" [摘要:智能] OR "患者为中心的临床研究网络" [摘要:智能] OR "PCORI" [摘要:智能] OR "患者导向医疗效果研究所" [摘要:智能] OR "以患者为中心结局研究机构" [摘要:智能] OR "患者导向医疗质量研究所" [摘要:智能] OR "以患者为中心的临床结局研究中心" [摘要:智能] OR "HL7" [摘要:智能] OR "美国卫生信息传输标准" [摘要:智能] OR "FHIR" [摘要:智能] OR "快速医疗互操作资源" [摘要:智能] OR "快速医疗互操作性资源" [摘要:智能] OR "SCDM" [摘要:智能] OR "哨点通用数据模型" [摘要:智能] OR "Mini-Sentinel" [摘要:智能] OR "迷你哨点" [摘要:智能] OR "微哨点" [摘要:智能] OR "Vaccine Safety Datalink" [摘要:智能] OR "疫苗安全性数据库链接" [摘要:智能] OR "疫苗安全数据库网" [摘要:智能] OR "疫苗安全数据系统" [摘要:智能] OR "疫苗安全数据链" [摘要:智能] OR "疫苗安全性数据链" [摘要:智能] OR "疫苗安全性数据系统" [摘要:智能] OR "PEDSnet" [摘要:智能] OR "儿科学习型健康医疗系统" [摘要:智能] OR "儿科学习健康系统" [摘要:智能] OR "VAESCO" [摘要:智能] OR "疫苗不良事件监测和沟通" [摘要:智能] OR "疫苗不良事件监测沟通" [摘要:智能] OR "疫苗不良事件监测与通信" [摘要:智能] OR "疫苗不良事件监测和通信" [摘要:智能] OR "Asian Pharmacoepidemiology Network" [摘要:智能] OR "亚洲药物流行病学网络" [摘要:智能] OR "亚洲药物流行病学合作网" [摘要:智能] OR "亚洲药物流行病学协作网" [摘要:智能] OR "i2b2" [摘要:智能] OR "ConcePTION通用数据模型" [摘要:智能] OR "NORPreSS" [摘要:智能] OR "北欧妊娠药物安全性研究" [摘要:智能] OR "CRN-VDW" [摘要:智能] OR "癌症研究网络虚拟数据仓库" [摘要:智能] OR "HCSRN" [摘要:智能] OR "卫生保健系统研究网络" [摘要:智能] OR "CESR" [摘要:智能] OR "有效性和安全性研究中心" [摘要:智能] OR "HMORN" [摘要:智能] OR "健康维护组织研究网络" [摘要:智能]

"通用数据模型" [标题:智能] OR "OMOP" [标题:智能] OR "观测性医疗结果合作组织" [标题:智能] OR "观察医疗结果合作项目" [标题:智能] OR "观察医疗结果合作伙伴关系" [标题:智能] OR "观察性医学产出合作项目" [标题:智能] OR "OHDSI" [标题:智能] OR "观察健康数据科学和信息学" [标题:智能] OR "观察性健康数据科学和信息学" [标题:智能] OR "健康观测数据科学和信息学" [标题:智能] OR "观察性健康医疗数据科学与信息学" [标题:智能] OR "CDISC" [标题:智能] OR "临床数据交换标准协会" [标题:智能] OR "SDTM" [标题:智能] OR "研究数据制表模型" [标题:智能] OR "研究数据表格模型" [标题:智能] OR "研究数据列表模型" [标题:智能] OR "研究数据制表标准" [标题:智能] OR "CDASH" [标题:智能] OR "临床数据获取协调标准" [标题:智能] OR "临床数据采集标准" [标题:智能] OR "临床数据获取标准协调" [标题:智能] OR "临床数据获取的标准协调" [标题:智能] OR "PCORnet" [标题:智能] OR "以患者为中心的国家级临床研究网络" [标题:智能] OR "国家患者中心临床研究网络" [标题:智能] OR "患者为中心的临床研究网络" [标题:智能] OR "PCORI" [标题:智能] OR "患者导向医疗效果研究所" [标题:智能] OR "以患者为中心结局研究机构" [标题:智能] OR "患者导向医疗质量研究所" [标题:智能] OR "以患者为中心的临床结局研究中心" [标题:智能] OR "HL7" [标题:智能] OR "美国卫生信息传输标准" [标题:智能] OR "FHIR" [标题:智能] OR "快速医疗互操作资源" [标题:智能] OR "快速医疗互操作性资源" [标题:智能] OR "SCDM" [标题:智能] OR "哨点通用数据模型" [标题:智能] OR "Mini-Sentinel" [标题:智能] OR "迷你哨点" [标题:智能] OR "微哨点" [标题:智能] OR "Vaccine Safety Datalink" [标题:智能] OR "疫苗安全性数据库链接" [标题:智能] OR "疫苗安全数据库网" [标题:智能] OR "疫苗安全数据系统" [标题:智能] OR "疫苗安全数据链" [标题:智能] OR "疫苗安全性数据链" [标题:智能] OR "疫苗安全性数据系统" [标题:智能] OR "PEDSnet" [标题:智能] OR "儿科学习型健康医疗系统" [标题:智能] OR "儿科学习健康系统" [标题:智能] OR "VAESCO" [标题:智能] OR "疫苗不良事件监测和沟通" [标题:智能] OR "疫苗不良事件监测沟通" [标题:智能] OR "疫苗不良事件监测与通信" [标题:智能] OR "疫苗不良事件监测和通信" [标题:智能] OR "Asian Pharmacoepidemiology Network" [标题:智能] OR "亚洲药物流行病学网络" [标题:智能] OR "亚洲药物流行病学合作网" [标题:智能] OR "亚洲药物流行病学协作网" [标题:智能] OR "i2b2" [标题:智能] OR "ConcePTION通用数据模型" [标题:智能] OR "NORPreSS" [标题:智能] OR "北欧妊娠药物安全性研究" [标题:智能] OR "CRN-VDW" [标题:智能] OR "癌症研究网络虚拟数据仓库" [标题:智能] OR "HCSRN" [标题:智能] OR "卫生保健系统研究网络" [标题:智能] OR "CESR" [标题:智能] OR "有效性和安全性研究中心" [标题:智能] OR "HMORN" [标题:智能] OR "健康维护组织研究网络" [标题:智能]
