# Supplementary material for: Pharmacoepidemiologic Research Based on Common Data Models: Systematic Review and Bibliometric Analysis
Source: JMIR Med Inform. 2025 Jul 28;13:e72225. doi: 10.2196/72225 (PMC12303556; doi:10.2196/72225)
Supplement: Multimedia Appendix 2 [file medinform-v13-e72225-s002.docx]

**Top 10 most-cited articles in pharmacoepidemiologic research based on CDMs through 2024.**

| **Rank** | **Author-Year** | **Journal** | **Citation counts** | **CDM type** | **Center counts** | **Country** | **Study exposure** | **Study outcome** |
| --- | --- | --- | --- | --- | --- | --- | --- | --- |
| 1 | N.P. Klein 2021 | JAMA | 391 | VSD | 8 | USA | COVID-19 Vaccine | Severe Adverse Events |
| 2 | Michael M. McNeil 2016 | Journal of Allergy and Clinical Immunology | 249 | VSD | 9 | USA | Influenza Vaccine, Hepatitis B Vaccine, Varicella Vaccine, MMR Vaccine, HPV Vaccine, DTaP Vaccine, Hepatitis A Vaccine, Polio Vaccine, Pneumococcal Vaccine, Rabies Vaccine, Herpes Zoster Vaccine | Anaphylaxis |
| 3 | W. Katherine Yih 2014 | New England Journal of Medicine | 221 | Mini-Sentinel | 3 | USA | Rotavirus Vaccine | Risk of Intussusception |
| 4 | Kari Bohlke 2003 | Pediatrics | 219 | VSD | 4 | USA | MMR Vaccine, Hepatitis B Vaccine, Pertussis Vaccine | Anaphylaxis |
| 5 | Nicola P. Klein 2010 | Pediatrics | 213 | VSD | 8 | USA | MMRV Vaccine | Febrile Seizures |
| 6 | George Hripcsak 2016 | PNAS | 180 | OMOP | 11 | USA | Antidiabetic Drugs, Antihypertensive Drugs, Antidepressants | Treatment Pathways |
| 7 | Julianne Gee 2011 | Vaccine | 172 | VSD | 7 | USA | HPV Vaccine | Guillain-Barré Syndrome, Stroke, Venous Thromboembolism, Appendicitis, Seizures, Syncope, Allergic Reaction, and Anaphylaxis |
| 8 | Elyse O. Kharbanda 2014 | JAMA | 162 | VSD | 2 | USA | DTaP Vaccine | Preterm Birth, Small for Gestational Age Infants |
| 9 | Sengwee Toh 2012 | Arch Intern Med | 158 | Mini-Sentinel | 17 | USA | Antihypertensive Drugs | Angioedema |
| 10 | Eric S. Weintraub 2014 | The New England Journal of Medicine | 155 | VSD | 6 | USA | Rotavirus Vaccine | Intussusception |

CDM: Common Data Model; VSD: Vaccine Safety Datalink; OMOP: Observational Medical Outcomes Partnership; USA: United States of America; COVID-19 vaccine: Coronavirus Disease 2019 vaccine; MMR vaccine: Measles, Mumps, and Rubella vaccine; HPV vaccine: Human Papillomavirus vaccine; DTaP vaccine: Diphtheria, Tetanus, and Acellular Pertussis vaccine.
