# Supplementary material for: Pharmacoepidemiologic Research Based on Common Data Models: Systematic Review and Bibliometric Analysis
Source: JMIR Med Inform. 2025 Jul 28;13:e72225. doi: 10.2196/72225 (PMC12303556; doi:10.2196/72225)
Supplement: Multimedia Appendix 3 [file medinform-v13-e72225-s003.docx]

**Comparison of Study Characteristics Between Articles Included and Not Included in the WoSCC.**

| **Variable** | **Non-WoSCC(N=23)** | **WoSCC (N=285)** | ***p* value** |
| --- | --- | --- | --- |
| Sample size (median, IQR) | 46,410 (17,456, 550,085) | 303,264 (22,069, 1,574,524) | 0.156 |
| Data from the United States (n, %) | 18 (78.26%) | 211 (74.04%) | 0.843 |
| Research exposure (n, %) |  |  | 0.736 |
| Drug | 11 (47.83%) | 121 (42.46%) |  |
| Vaccine | 12 (52.17%) | 161 (56.49%) |  |
| Medical device | 0 (0.00%) | 3 (1.05%) |  |
| Types of CDMs (n, %) |  |  | 0.086 |
| OMOP | 10 (43.48%) | 66 (23.16%) |  |
| VSD | 7 (30.43%) | 153(53.68%) |  |
| Sentinel/mini-Sentinel | 2 (8.70%) | 29 (10.18%) |  |
| Others | 4(17.39%) | 37(12.98%) |  |
| Research Directions (n, %) |  |  | 0.924 |
| Safety | 18 (78.26%) | 199 (69.82%) |  |
| Effectiveness | 1 (4.35%) | 14 (4.91%) |  |
| Utilization | 3 (13.04%) | 47 (16.49%) |  |
| Others | 1 (4.35%) | 25 (8.77%) |  |
| Including sensitive analysis (n, %) | 4 (17.39%) | 86(30.71%) | 0.237 |
| Including subgroup analysis (n, %) | 15 (65.22%) | 129 (45.42%) | 0.107 |
| Including reporting guideline (n, %) | 0 (0.00%) | 16 (5.61%) | 0.619 |

WoSCC: Web of Science Core Collection, IQR: Interquartile Range; CDM: Common Data Model; OMOP: Observational Medical Outcomes Partnership; VSD: Vaccine Safety Datalink.
