# Supplementary material for: Pharmacoepidemiologic Research Based on Common Data Models: Systematic Review and Bibliometric Analysis
Source: JMIR Med Inform. 2025 Jul 28;13:e72225. doi: 10.2196/72225 (PMC12303556; doi:10.2196/72225)
Supplement: Multimedia Appendix 4 [file medinform-v13-e72225-s004.docx]

**Top 10 authors with most articles in pharmacoepidemiologic research based on CDMs** **through 2024.**

| **Rank** | **Author** | **Publication counts** | **Country** | **H-index** | **Total Citations** | **Institutions** |
| --- | --- | --- | --- | --- | --- | --- |
| 1 | KLEIN NP | 69 | USA | 29 | 2930 | Kaiser Permanente Vaccine Study Center |
| 2 | DALEY MF | 53 | USA | 16 | 1086 | Institute for Health Research, Kaiser Permanente Colorado  Department of Pediatrics |
| 3 | NALEWAY AL | 48 | USA | 22 | 1294 | The Center for Health Research, Kaiser Permanente Northwest |
| 4 | JACKSON LA | 41 | USA | 18 | 1214 | Group Health Research Institute |
| 5 | NORDIN JD | 41 | USA | 25 | 1601 | HealthPartners Institute for Education and Research, and Minneapolis VA Health Care System and University of Minnesota |
| 6 | HAMBIDGE SJ | 40 | USA | 22 | 1963 | Institute for Health Research, Kaiser Permanente Colorado |
| 7 | GLANZ JM | 39 | USA | 19 | 1588 | Institute for Health Research, Kaiser Permanente Colorado |
| 8 | WEINTRAUB ES | 39 | USA | 21 | 1948 | Centers for Disease Control and Prevention, Immunization Safety Office |
| 9 | KHARBANDA EO | 38 | USA | 21 | 1878 | HealthPartners Institute for Education and Research |
| 10 | WEINTRAUB E | 37 | USA | 19 | 1265 | Immunization Safety Office, Centers for Disease Control and Prevention |

CDM: Common Data Model; USA: United States of America.
