# Supplementary material for: Pharmacoepidemiologic Research Based on Common Data Models: Systematic Review and Bibliometric Analysis
Source: JMIR Med Inform. 2025 Jul 28;13:e72225. doi: 10.2196/72225 (PMC12303556; doi:10.2196/72225)
Supplement: Multimedia Appendix 5 [file medinform-v13-e72225-s005.docx]

**Top 10 institutions with most articles in pharmacoepidemiologic research based on CDMs** **through 2024.**

| **Rank** | **Institution** | **Counts** | **Country** |
| --- | --- | --- | --- |
| 1 | KAISER PERMANENTE | 505 | USA |
| 2 | CENTERS FOR DISEASE CONTROL AND PREVENTION - USA | 149 | USA |
| 3 | HARVARD UNIVERSITY | 124 | USA |
| 4 | HARVARD PILGRIM HEALTH CARE | 96 | USA |
| 5 | HARVARD MEDICAL SCHOOL | 95 | USA |
| 6 | US FOOD AND DRUG ADMINISTRATION (FDA) | 61 | USA |
| 7 | SEOUL NATIONAL UNIVERSITY (SNU) | 60 | Korea |
| 8 | HALLYM UNIVERSITY | 57 | Korea |
| 9 | DENVER HEALTH MEDICAL CENTER | 42 | USA |
| 10 | UNIVERSITY OF CALIFORNIA SYSTEM | 40 | USA |

CDM: Common Data Model; USA: United States of America. Each author's affiliation is counted individually.
