# Supplementary material for: Pharmacoepidemiologic Research Based on Common Data Models: Systematic Review and Bibliometric Analysis
Source: JMIR Med Inform. 2025 Jul 28;13:e72225. doi: 10.2196/72225 (PMC12303556; doi:10.2196/72225)
Supplement: Multimedia Appendix 6 [file medinform-v13-e72225-s006.docx]

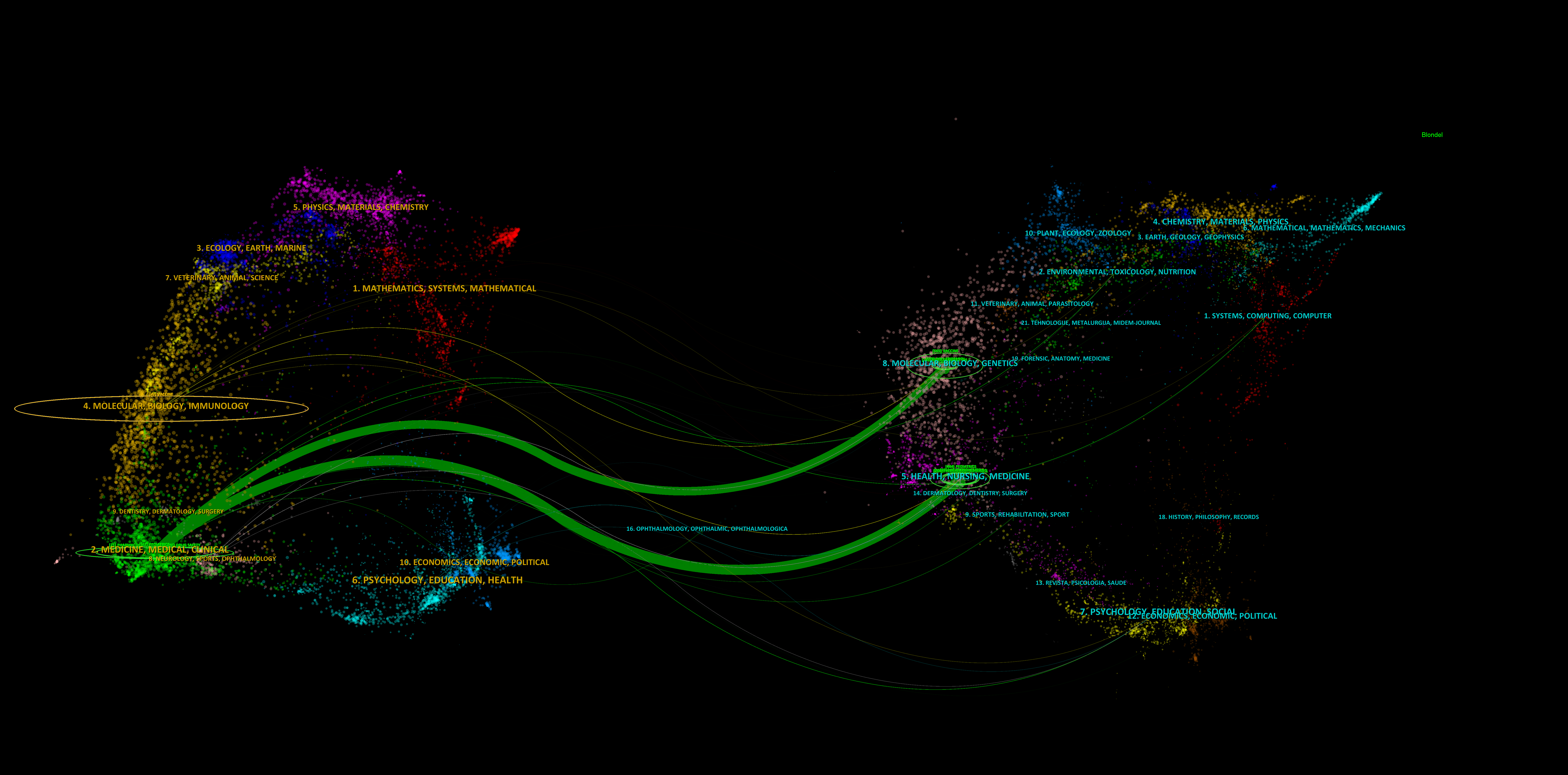


**Dual-map overlay of journal citation trajectories in CDM-based pharmacoepidemiologic research.**

This map illustrates the interdisciplinary citation landscape, with the left side representing the distribution of journals where the citing articles were published, and the right side representing the journals that were most frequently cited. The colored curves between the two sides indicate the main citation paths, showing how knowledge flows across disciplines.
